# FUS-mediated blood–brain barrier disruption for delivering anti-Aβ antibodies in 5XFAD Alzheimer’s disease mice

**DOI:** 10.1007/s40477-023-00805-4

**Published:** 2023-07-29

**Authors:** Anastasia Antoniou, Marios Stavrou, Nikolas Evripidou, Elena Georgiou, Ioanna Kousiappa, Andreas Koupparis, Savvas S. Papacostas, Kleopas A. Kleopa, Christakis Damianou

**Affiliations:** 1 Department of Electrical Engineering, Computer Engineering, and Informatics, Cyprus University of Technology, Limassol, Cyprus; 2 Department of Neurobiology, The Cyprus Institute of Neurology and Genetics, Nicosia, Cyprus; 3 Department of Neuroscience, The Cyprus Institute of Neurology and Genetics, Nicosia, Cyprus

**Keywords:** Alzheimer’s disease, Ultrasound, BBB, Anti-Αβ, Antibody, Mice

## Abstract

**Purpose:**

Amyloid-β (Aβ) peptides, the main component of amyloid plaques found in the Alzheimer's disease (AD) brain, are implicated in its pathogenesis, and are considered a key target in AD therapeutics. We herein propose a reliable strategy for non-invasively delivering a specific anti-Aβ antibody in a mouse model of AD by microbubbles-enhanced Focused Ultrasound (FUS)-mediated Blood–brain barrier disruption (BBBD), using a simple single stage MR-compatible positioning device.

**Methods:**

The initial experimental work involved wild-type mice and was devoted to selecting the sonication protocol for efficient and safe BBBD. Pulsed FUS was applied using a single-element FUS transducer of 1 MHz (80 mm radius of curvature and 50 mm diameter). The success and extent of BBBD were assessed by Evans Blue extravasation and brain damage by hematoxylin and eosin staining. 5XFAD mice were divided into different subgroups; control (*n = *1), FUS + MBs alone (*n = *5), antibody alone (*n = *5), and FUS + antibody combined (*n = *10). The changes in antibody deposition among groups were determined by immunohistochemistry.

**Results:**

It was confirmed that the antibody could not normally enter the brain parenchyma. A single treatment with MBs-enhanced pulsed FUS using the optimized protocol (1 MHz, 0.5 MPa in-situ pressure, 10 ms bursts, 1% duty factor, 100 s duration) transiently disrupted the BBB allowing for non-invasive antibody delivery to amyloid plaques within the sonicated brain regions. This was consistently reproduced in ten mice.

**Conclusion:**

These preliminary findings should be confirmed by longer-term studies examining the antibody effects on plaque clearance and cognitive benefit to hold promise for developing disease-modifying anti-Aβ therapeutics for clinical use.

## Introduction

Blood–Brain-Barrier (BBB) protects the central nervous system (CNS) from drugs and toxins. It is composed of microvascular endothelial cells. Tight junctions (TJs) are formed between these cells, with several transporters regulating the influx and efflux of compounds, such as nutrients and small peptides [1]. Generally, paracellular permeability is limited to substances with a molecular weight up to 400–500 Da, thus prohibiting the delivery of most therapeutic agents into the brain [2]. The highly selective nature of BBB is the main obstacle against the application of potential disease-modifying therapies for diseases of the CNS, including neurodegenerative diseases such as the Alzheimer's disease (AD) [3, 4]. Accordingly, drug delivery into the brain tissue has been a major challenge for researchers over a long period.

It is by now generally accepted that pulsed FUS in synergy with microbubbles (MBs) can cause temporal BBBD by causing alterations in the cell-to-cell interactions and endothelial cell cytoskeleton. In fact, MBs-enhanced FUS was shown to loosen the endothelial cell tight junctions (TJs) through a mechanism known as cavitation [5, 6]. The junctions’ disruption is mainly attributed to changes in the level of related trans- and peripheral membrane proteins [7]. In addition, FUS treatment was found to cause stimulation of transcytosis, sonoporation of the vascular endothelium, and increase in the paracellular diffusion due to the TJs disruption [7]. FUS can further cause disruption of drug efflux by temporally suppressing the expression of the permeability-glycoprotein (Pgp) [8].

BBBD by pulsed FUS in the presence of gaseous MBs has emerged as a feasible method of delivering large molecules normally hampered by the BBB to the brain. This strategy has been confirmed by numerous preclinical studies to enhance the penetration of therapeutic agents, such as therapeutic peptides, genes, and antibodies into the CNS of non-transgenic and transgenic mouse models of neurological diseases, with an increasing number of clinical trials exploring clinical utility [9–13]. Typically, initial evidence of the success and extend of BBBD is obtained by contrast-enhanced MRI and the well-known Evans Blue (EB) dye method [10, 11, 13].

AD is the prevalent neurodegenerative disorder and cause of dementia and is characterized by the presence of intracellular neurofibrillary tangles and extracellular amyloid plaques owing to Amyloid β peptides (Aβ) aggregation [14, 15]. Available treatments are not curative but may slow disease progression and alleviate symptoms. Given the urgent demand for disease-modifying therapies, the development of FUS therapeutics for AD receives remarkable research interest.

The ability of MBs-enhanced FUS without exogenous agents to reduce the Aβ pathology has been well demonstrated [16–18]. A single trans-skull MRgFUS treatment was shown to increase the levels of endogenous immunoglobulins (IgM and IgG) in the cortex of the TgCRND8 mouse model [16]. FUS-mediated endogenous antibody delivery and glia cells activation were considered as the mechanisms responsible for the observed plaque burden reduction [16]. Later, Shen et al. [17] reported that FUS in synergy with MBs applied twice a week for 6 weeks triggered behavioral changes and improved the spatial memory of triple transgenic AD mice. These changes were associated with reduced Aβ pathology and tau phosphorylation, as well as improved neuronal health of the sonicated hippocampus compared to the sham group.

The positive effects of FUS in the mitigation of AD pathological features can be enhanced by administrating exogenous therapeutic agents. According to a study by Hsu et al. [19], the effects of FUS on plaque reduction were enhanced using a specific inhibitor of the glycogen synthase kinase-3 (GSK-3); a key molecule in the onset of AD. Administration of this inhibitor in APPswe/PSEN1-dE9 transgenic mice prior to MBs-enhanced FUS reduced the Aβ plaque synthesis by suppressing the GSK-3 protein activity [19]. Another study targeted an Aβ peptide species deposited in AD brain termed Pyroglutamate-3 Aβ (pGlu-3 Aβ) [20]. The FUS-mediated administration of an anti-pGlu-3 Aβ vaccine was found to promote plaque clearance and partial protection from cognitive decline in APPswe/PS1ΔE9 mice [20]. Others attempted to support neuronal health as a measure for disease mitigation [21]. The repeated MRgFUS-mediated delivery of a pharmacological agent termed D3 (TrkA agonist) that promotes neuronal function was found to impart numerous therapeutic effects, including enhanced hippocampal neurogenesis and positive cognitive effects in TgCRND8 AD mice [21].

Several studies aimed to investigate the efficiency of FUS-mediated BBBD to facilitate the supply of large disease-specific antibodies in the brain and the resultant therapeutic effects. The feasibility of delivering an anti-Ab antibody called BAM-10 into the brain of the TgCRND8 mouse model using transcranial MRgFUS and reducing the plaque pathology has been demonstrated by Jordao et al. [22]. FUS-induced BBBD was also shown to facilitate the supply of an anti-pyroglutamate-3 Aβ monoclonal antibody (mAb) called 07/2a in the brain of aged APP/PS1dE9 transgenic mice [23]. Sun et al. [24] further demonstrated that three successive weekly treatments with the 07/2a mAb combined with FUS resulted in a faster improvement of spatial learning and memory of a higher percentage of aged APP/PS1dE9 mice compared to the mice group receiving only antibody.

Another anti-Αβ antibody tested for its efficacy to improve cognition in AD mice is the Aducanumab. Leinenga et al. [25] compared the effects of this antibody when administered alone or in synergy with MBs-enhanced scanning ultrasound in APP23 AD mice. The combined approach resulted in a five-fold increase in the antibody amount compared to the non-sonicated mice a few days post-treatment and significant improvement in spatial memory. Notably, Aducanumab is the first therapeutic agent to be tested in combination with FUS in AD patients in a phase I ongoing clinical trial [26].

The Aβ (1–40) antibody targets the amyloid peptides Aβ (1–40) that represent the most abundant Aβ isoform in the AD brain [27]. The FUS-mediated delivery of the specific antibody was previously tested in a very small mice population (*n = *3) [28]. A three-fold increase in fluorescence intensity of the antibody staining was observed in the brain regions treated with MBs-enhanced MRgFUS in comparison with the non-sonicated regions, with hematoxylin and eosin (H&E) staining providing evidence of hemorrhages in the sonicated brain tissue [28]. While this study provides promising results on FUS-mediated enhanced Aβ (1–40) antibody delivery, further experiments in a larger mouse population are needed to confirm these early findings and optimize the therapeutic protocol for safe and efficient Aβ (1–40) antibody delivery.

In this study, we aimed to evaluate whether the application of FUS in synergy with MBs using an in-house manufactured manual positioning device comprising a single element FUS transducer of 1 MHz can facilitate the penetration of the Aβ (1–40) antibody into the brain of 5XFAD transgenic mice. We initially attempted to define the sonication protocol for safe and efficient BBBD. The success and extent of BBBD was assessed by EB extravasation while brain damage was assessed by H&E staining. We then examined the capability of the Aβ (1–40) antibody to consistently enter the brain parenchyma when administered alone and prior to MBs-enhanced FUS using the optimized protocol in a large 5XFAD mice group.

## Materials and methods

All mice experiments were carried out at the premises of the Cyprus Institute of Neurology and Genetics under national guidelines and protocols authorized by the Veterinary Services of Cyprus under the study license CY/EXP/PR.L05/2021.

### FUS system

**Fig. 1:**
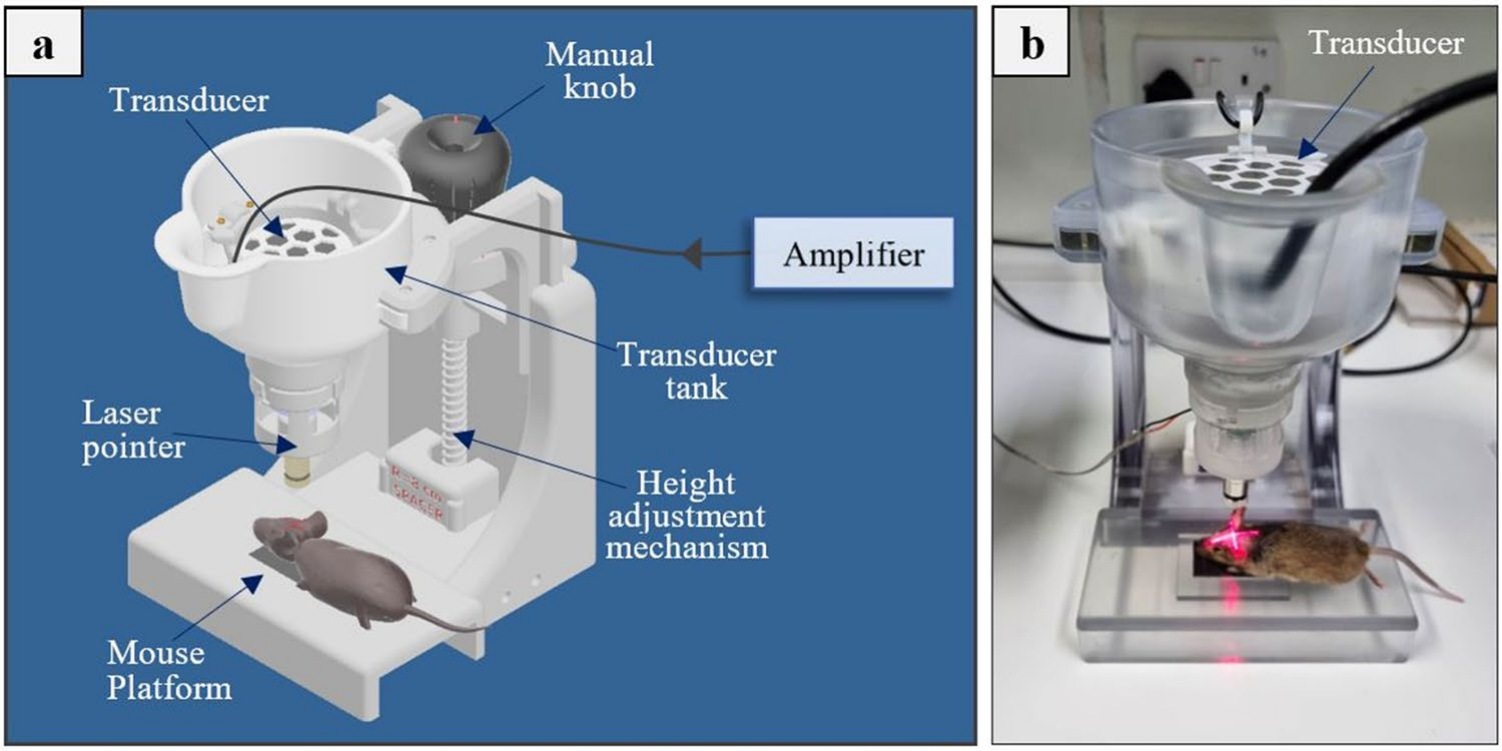
**a** CAD drawing of the 1-DOF manual positoning device comprising a FUS transducer of 1 MHz with a mouse positioned on the dedicated platform, **b** Indicative photo from experiment

FUS was delivered using a manual positioning system [29] comprising a single element, spherically focused, ultrasound transducer (Piezo Hannas, Wuhan, Hubei, China, 1 MHz central frequency, 80 mm radius of curvature, 50 mm diameter, and 32.5% acoustic efficiency) tuned to an RF amplifier (AG 1016, AG series, T&G Power conversion Inc., Rochester, NY). This system was specially designed to facilitate transcranial FUS studies in rodents. The transducer is hosted in a conical water tank whose bottom opening is sealed with a silicone membrane. The tank can be moved vertically via a manual positioning mechanism coupled to the mouse platform to attach to the mouse head via a top to bottom approach. A laser pointer accessory was implemented into the system to facilitate consistent targeting among experiments. The positioning device and animal placement on the dedicated platform can be seen in Fig. 1.

### Protocol optimization for efficient and safe Blood–brain barrier disruption

Thirty-two (32) WT B6/SJL mice were used for protocol calibration/optimization. Intraperitoneal injection of Avertin (Sigma Aldrich, St. Louis, Missouri, United States) was used to cause rapid and deep anesthesia in mice and ensure no suffering. The dose of Avertin was weight-dependent for each animal (20 μL/g). The hair was removed from the mouse head using a commercial hair removal cream (Veet Hair Removal cream). Retro-orbital injection was then used to deliver a mixture of 5 μL of SonoVue^®^ MBs (Bracco Imaging, Turin, Italy, 2 × 10^8^ microbubbles/ mL suspension) along with 5 mL/kg of 3% w/v EB solution (Sigma, St. Louis, MO, USA). Anesthetized animals were positioned in prone position on the platform as shown in Fig. 1. The tank was filled with degassed, deionized water and US coupling gel (Quick-Eco Gel, AB Medica group S.A., Barcelona, Spain) was applied on the mouse head to achieve efficient acoustic coupling. The position of the mouse was adjusted so that the FUS beam was targeted on the left hemisphere centrally with the assistance of the laser system. All mice received a single sonication within 3–4 min following the injection of MBs and EB using 1 MHz pulsed FUS of 10 ms bursts at a duty factor (DF) of 1% for a total duration of 100 s.

For protocol calibration purposes, electric power values of 20 to 70 W were tested (10 W step; 6 groups of 5 mice each). The relevant acoustic power ranged from 6.5 to 22.8 W, corresponding to in situ focal acoustic pressure in the range of 0.3–0.6 MPa. The output acoustic power was estimated based on the acoustic efficiency of the transducer of 32.5%. The respective focal pressures were initially determined in water using a needle hydrophone (HNC, ONDA, Sunnyvale, CA, USA) placed at the focal distance of the transducer. *In-situ* pressures were then calculated accounting for the transmission loss through the mouse skull. The transmission coefficient of a skull sample was measured according to the well-established through-transmission immersion technique [30] at the operating frequency of the transducer of 1 MHz. One mouse received only EB and one neither EB nor FUS, thus serving as the control mice.

Mice were sacrificed by transcardial perfusion with saline followed by 4% paraformaldehyde (PFA) 40 min post-treatment. This time period is well within the 4-h window that the BBB was found to maintain open after FUS. Therefore it was considered sufficient for successful entry of EB into the brain, but also to allow for acute FUS-induced physiological responses to be resolved [31]. The brain tissue was then collected and preserved in paraformaldehyde (4%) and then sucrose (20%) diluted in Phosphate Buffer (0.1%) according to our protocol. Brain sections were prepared for fluorescence imaging. Slides containing brain sections were visualized using a Nikon eclipse-Nἱ (Tokyo, Japan) fluorescence microscope to visualize EB extravasation and determine the BBB-opened region.

### Trans-BBB Aβ (1–40) antibody delivery in a mouse model of AD

#### Animals

5XFAD transgenic mice recapitulating major pathological features of AD were utilized. 5XFAD mice were bred as single transgenics. Male 5XFAD mice were crossed with female SJL/B6 F1 mice to give hemizygous or wild-type offspring's, which were used for the purpose of the study. The pathologic phenotype of this mouse model consists of gliosis, amyloid plaques, neurodegeneration, memory deficits (at 4–5 months), as well as intraneuronal Aβ and neuron loss. Beginning at 8 weeks of age, amyloid deposition and gliosis become increasingly widespread, especially in the deep cortical layers and subiculum.

#### Experimental design

5XFAD transgenic mice of 5-months of age (*n = *21) were used to test the feasibility and efficacy of FUS-mediated delivery of the Aβ (1–40) antibody (150 kDa, Anti-β-Amyloid Protein (1–40) antibody produced in rabbit whole antiserum, A8326, Sigma Aldrich, 3050 Spruce Street, Saint Louis, MO 63103, USA) into the brain. The ability of the antibody to pass through the BBB and bind to the Aβ plaques when administered alone and in combination with FUS was investigated using a constant antibody amount of 50 μL (2.85 mg), which is the half quantity of the previously tested antibody dose [28].

Twenty one (21) mice were divided into 4 sub-groups: A. Staining with the Aβ (1–16) and Aβ (1–40) antibodies without injected antibody or FUS + MBs to confirm the presence of amyloid plaques in the cortex (referred to as control, *n = *1), B. Saline (50 μL) administration followed by FUS + MB-induced BBB opening (referred to as saline; *n = *5), C. Aβ (1–40) antibody (50 μL) administration alone (referred to as antibody, *n = *5), and D. Aβ (1–40) antibody (50 μL) administration followed by FUS + MB-induced BBB opening (referred to as FUS + MBs plus Ab; *n = *10).

**Fig. 2:**
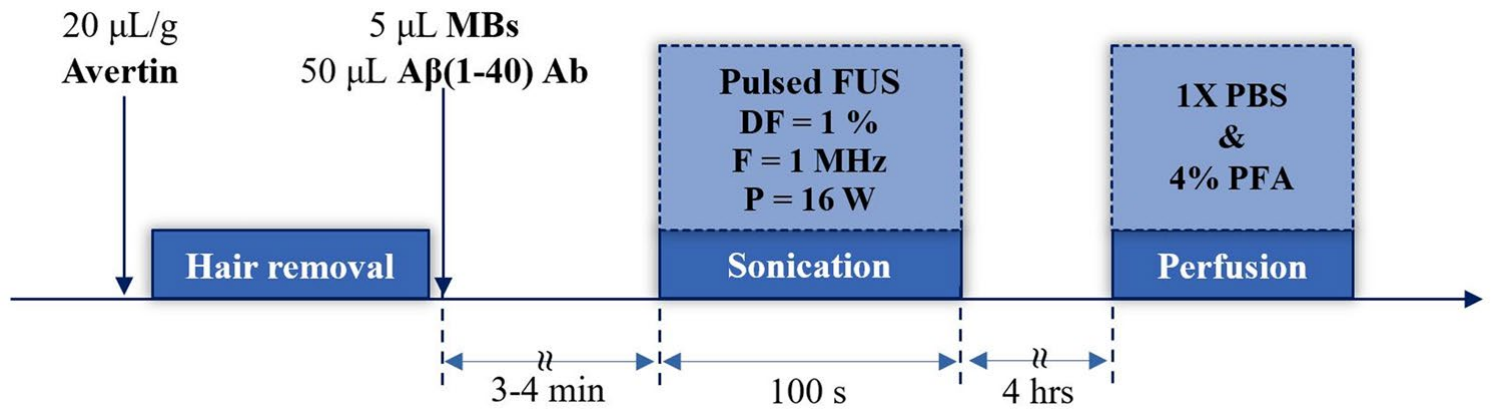
Protocol timeline for FUS-mediated Aβ (1–40) antibody delivery in 5XFAD mice

The anesthesia protocol and treatment timeline were similar to that used for the calibration study. The Aβ (1–40) antibody was delivered instead of the EB dye via retro-orbital injection along with the MBs. Based on the data gathered from the protocol optimization study, an acoustic power of 16 W (in situ focal acoustic pressure of 0.5 MPa) was considered optimum and used in this experimental part while the rest sonication parameters remained the same. The treatment protocol is summarized in the diagram of Fig. 2. Note that following FUS, a time window of 4 h was left before mice sacrifice.

#### Mouse sacrifice and tissue preparation

Transcardial perfusion was used to clear blood and preserve the brain for immunostaining analysis. Following perfusion, the mouse head was dissected, and the skull was carefully removed using scissors and forceps, exposing the brain. The brain was washed in Phosphate Buffer Saline (PBS) and then placed for 2 h in 4% Paraformaldehyde (PFA) solution. Subsequently, it was again washed with PBS and placed into 20% sucrose solution (diluted in Phosphate Buffer 0.1 M) overnight at 4 °C for cryoprotection prior to embedding and freezing. For tissue embedding, the cryomould containing the brain tissues, was filled with OCT, and placed into acetone-dry ice bath. Finally, the frozen OCT containing the brain tissue was removed from the cryomould and stored in a − 80 °C freezer.

#### Immunohistochemistry

Double immunostaining of coronal brain Sects. (16 brain sections / mouse) was performed to determine whether the injected Aβ (1–40) antibody passed the BBB and bound to Aβ plaques. Staining with the Aβ (1–16) antibody (6E10, green colour) was used to identify the amyloid plaques. The tissue was permeabilized by immersing the frozen sections in acetone for 10 min at − 20 °C. It was then washed three times with 1X PBS and blocking solution (5% Bovine Serum Albumin + 0.5% Triton X-100) was applied for 1 h on the sections at room temperature in a humidified chamber. The blocking solution was then removed and the primary antibody; anti-β-amyloid primary monoclonal 6E10 (1 mg/mL) (diluted in blocking solution, 1:400) was applied to the tissue sections and incubated overnight at 4 °C. The following day, the primary antibody was removed, and the tissue sections were washed three times with 1X PBS. The secondary antibodies; Fluorescein (FITC) goat anti-mouse (1.5 mg/mL), 1:100 and Alexa Fluor® 594 goat anti-rabbit (2 mg/mL), 1:500 (diluted in blocking solution) were next applied for 1 h at room temperature for the detection of the injected antibody in the examined brain tissue, followed by three washes with 1X PBS and incubation for 30 s with 4ʹ,6-diamidino-2-phenylindole (DAPI, Sigma-Aldrich) for nuclear staining. The tissues were washed two times for 5 min with 1X PBS, dried and mounted with mounting media in order to prepare them for microscopy.

#### H&E staining

We also checked the tissue integrity and the lack of hemorrhage with H&E staining for the tested acoustic pressures ranging from 0.3 to 0.6 MPa. Tissue sections in OCT were stained with Harris's haematoxylin (freshly filtered) for 3 min, and then washed with distilled water and stained with aqueous eosin for 6 min. Next, they were dehydrated in ascending concentrations of alcohol and cleared in xylene (70%, 95%, 100% × 2 and xylene × 3). Finally, the tissue slides were mounted with Dibutylphthalate Polystyrene Xylene (DPX).

## Results

### Protocol optimization for efficient and safe Blood–brain barrier disruption

**Fig. 3:**
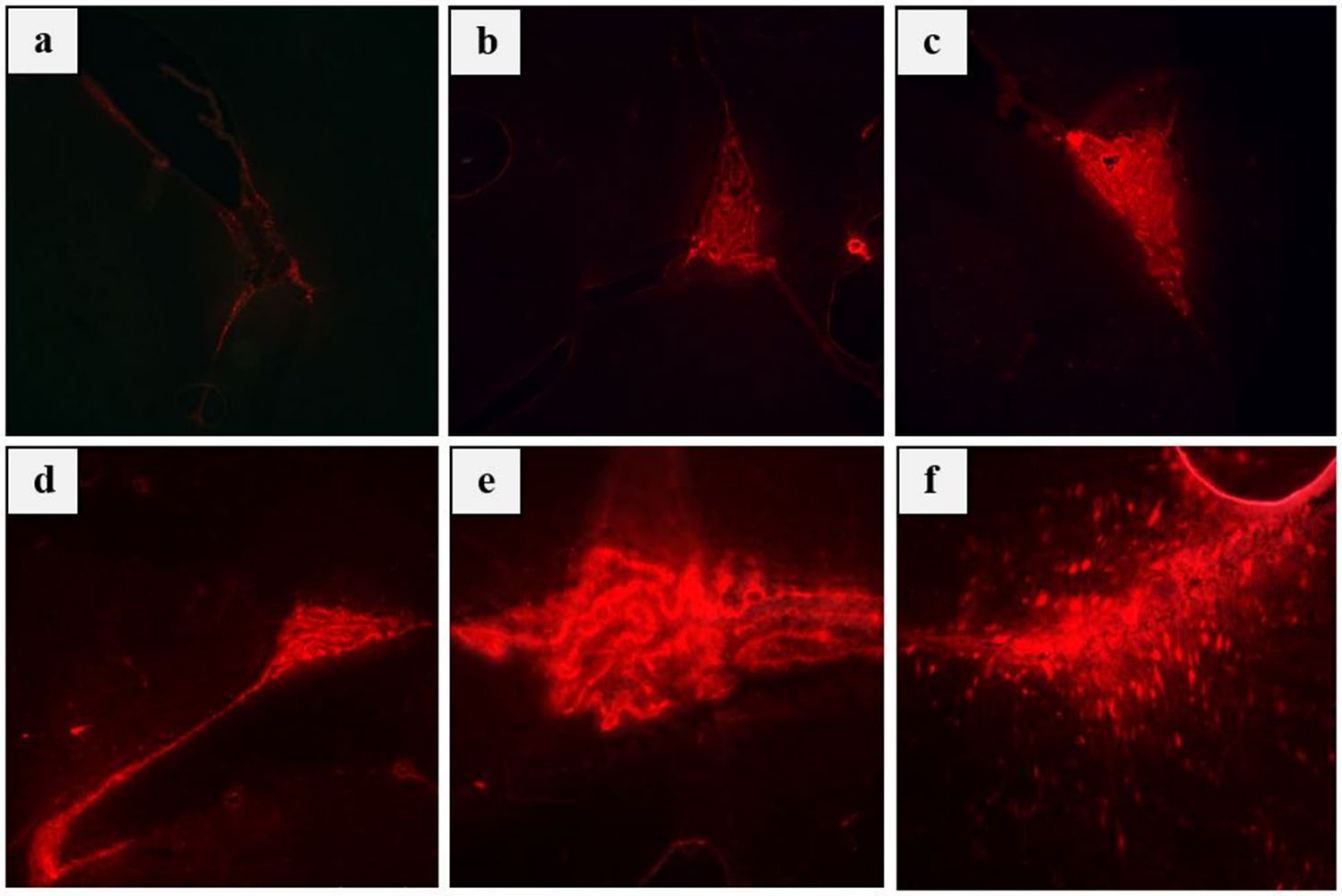
Fluorescence images (10 × magnification) of unstained brain sections at the level of the lateral ventricles of mice injected with EB:** a** No FUS, **b** FUS at 6.5 W, **c** FUS at 9.7 W, **d** FUS at 13 W, **e** FUS at 16 W, and **f** FUS at 19.5 W (acoustic power)

According to fluorescence microscopy all tested power levels in the range of 6.5–22.8 W (0.3−0.6 MPa in situ pressure) combined with 5 μL of MBs (for the specific sonication parameters employed) caused BBBD since increased fluorescence intensity of EB was observed compared to the control mouse (receiving only EB). Indicative fluorescence images for the various acoustic powers tested are presented in Fig. 3, revealing the power effect on the extent of EB extravasation. Note that a gradual increase of fluorescence intensity (indicating increase in the extent of BBBD) occurred as the power in the tested range was increased.

**Fig. 4:**
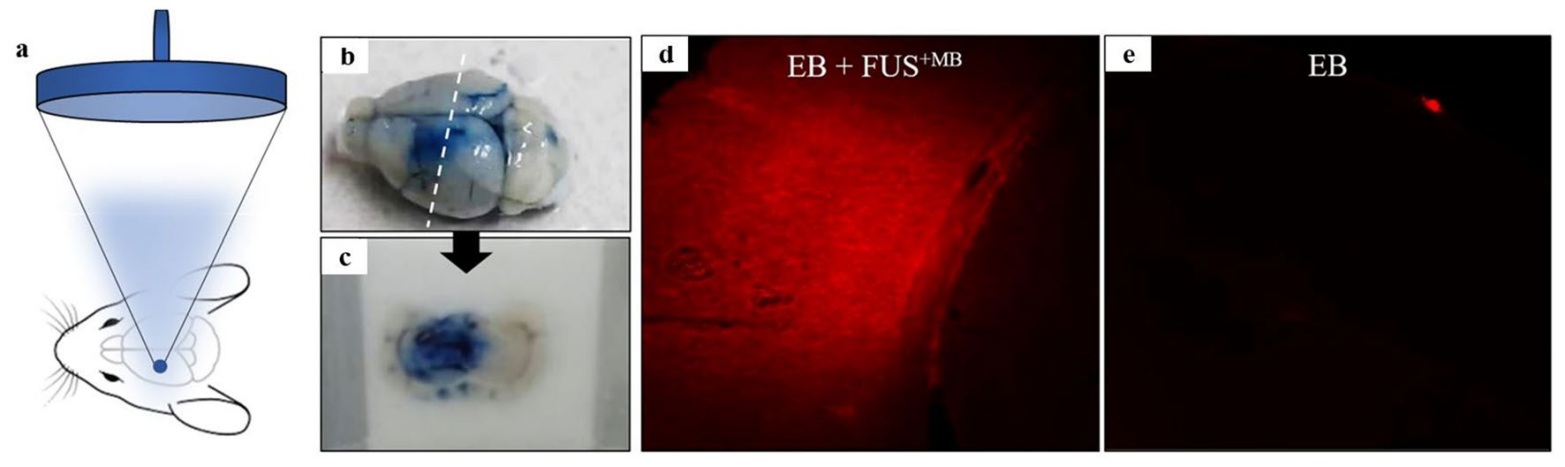
**a** FUS beam targeting centrally at the left hemisphere, **b** Freshly perfused excised mouse brain treated with the selected protocol (5 μL MBs and 16 W acoustic power), **c** brain section after fixation in OCT revealing the distribution pattern of EB extravasation, **d–e** Fluorescence images (5 × magnification) of unstained brain sections at the level of the cortex taken from perfused mice injected with EB and 5 μL MBs followed by sonication at 16 W (EB + FUS^+MB^) and injected with EB only (control)

**Fig. 5:**
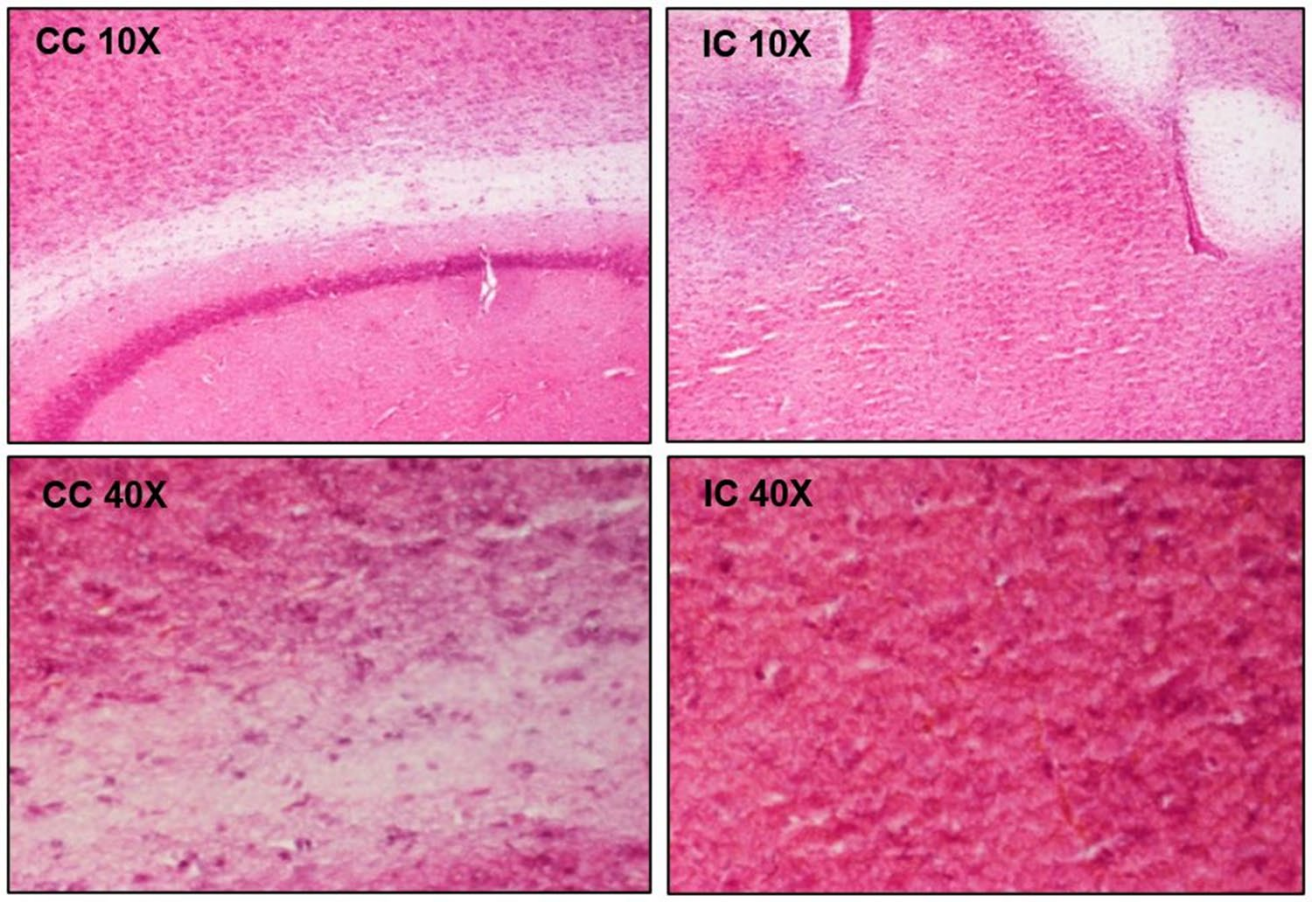
Representative photos (10 × and 40 × magnification) of H&E staining from mice treated with MBs-enhanced pulsed FUS at 16 W for two different brain areas; corpus callosum (CC) and inferior colliculus (IC)

The optimal power was selected as the one resulting in the highest EB leakage (ideally spread throughout the sonicated region) without causing any adverse effects on tissue and having consistent behavior among subjects. The power value of 16 W (0.5 MPa in situ pressure) showed consistent EB leakage in all examined brain regions and no evidence of damage and was thus selected for follow-up experiments in the AD mouse model. Figure 4 shows representative fluorescence microscopy images for the selected power taken from to cortex region. Photos of the freshly perfused excised brain of a mouse treated with the selected protocol and a brain section after fixation in OCT can be seen in Fig. 4b and c, respectively. Note that the EB dye was diffused throughout the entire left hemisphere that was sonicated. Figure 4d and e compare magnified fluorescence images of unstained brain sections at the level of the cortex between a non-sonicated mouse and a sonicated mouse (pulsed FUS with 10 ms burst length and 1% DF at 16 W for 100 s duration and 5 μL MBs) both injected with equal amount of EB solution (5 mL/kg of 3% w/v). Note that no leakage was observed in the brain of the control mouse (EB only), whereas FUS-induced BBBD resulted in high levels of EB dye covering the examined cortex area. Indicative histological slides from H&E examination for the selected acoustic power (16 W) from two different brain areas can be seen in Fig. 5. No difference between the FUS treated and control cases in terms of tissue integrity was observed and there was no evidence of hemorrhage in none of the tested brain regions.

### Trans-BBB Aβ (1–40) antibody delivery in a mouse model of AD

**Fig. 6:**
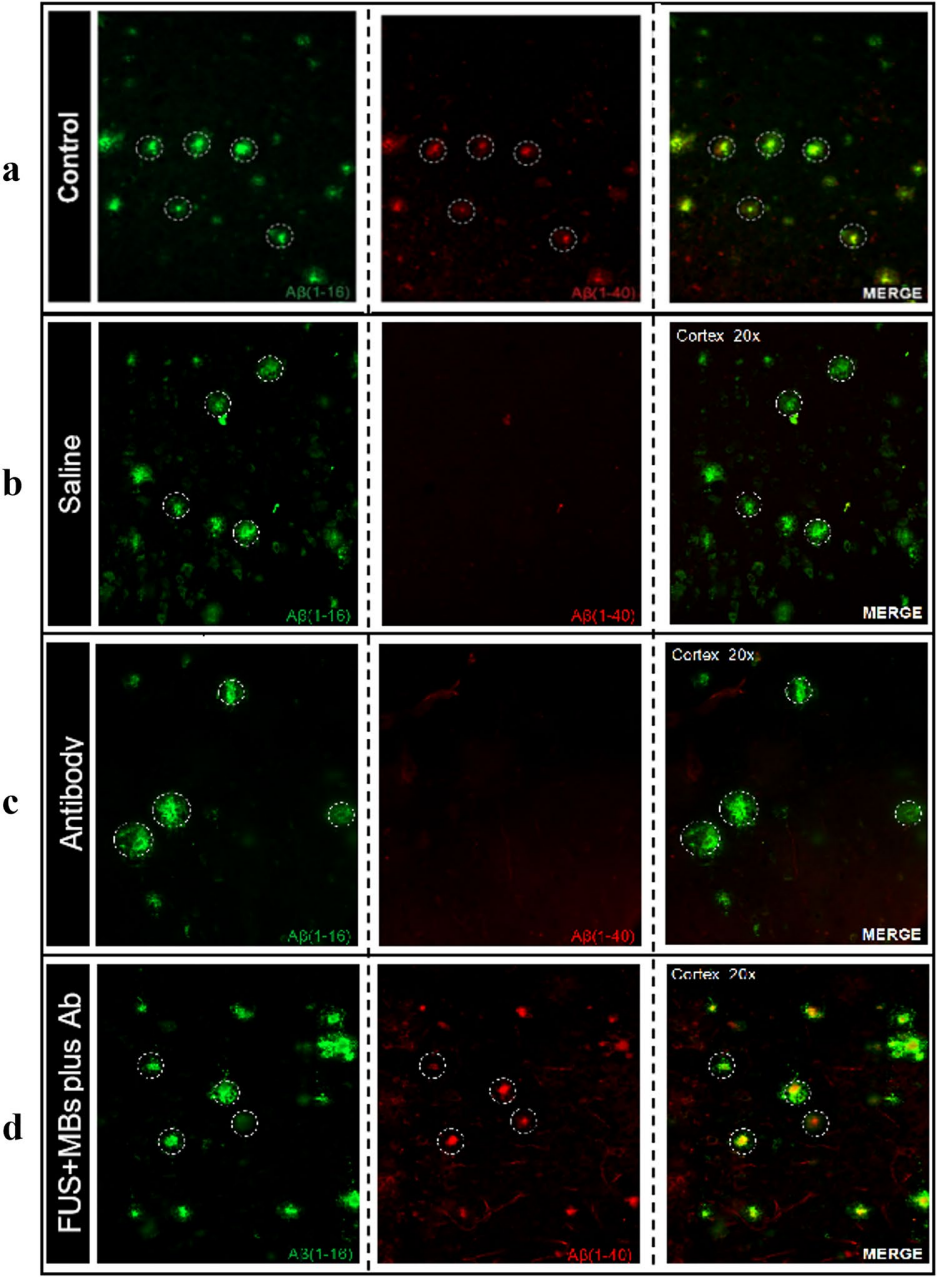
Immunohistochemistry analysis of brain tissue sections of 5xFAD mice. **a** Control staining; brain tissue without any injected antibody or FUS + MBs stained with Aβ (1–16) and Aβ (1–40) antibodies to confirm the presence of amyloid plaques (green & red) in the cortex. **b** Mouse injected with saline followed by FUS + MBs. **c** Mouse injected with the Aβ (1–40) antibody alone. **d** Mouse injected with 50 μL of Aβ (1–40) antibody followed by FUS + MBs. Amyloid plaques (green) were stained with Aβ (1–16)**.** The Aβ (1–40) antibody was stained red. Co-localization of antibodies (white circles) in the cortex of the FUS + MBs plus Ab group (MERGE) confirmed successful entry and binding of the Aβ (1–40) with amyloid plaques (Sonication parameters: *f = *1 MHz, burst length = 10 ms, D*F = *1%, acoustic power = 16 W, and sonication duratio*n = *100 s)

**Fig. 7:**
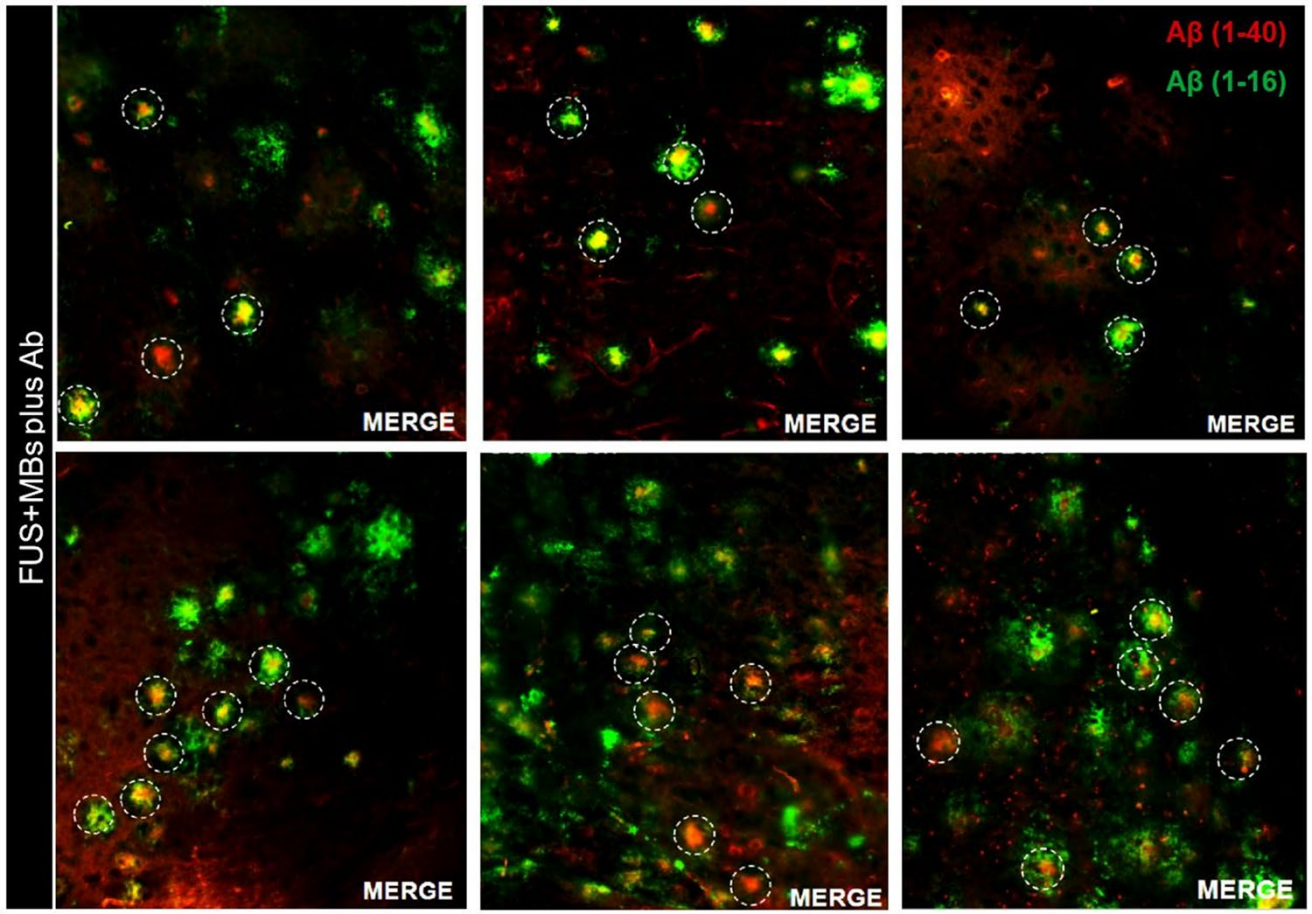
Immunohistochemistry analysis of brain tissue sections of 5XFAD mice injected with 50 μL of Aβ (1–40) and 5 μL MBs followed by pulsed FUS (*f = *1 MHz, burst length = 10 ms, D*F = *1%, acoustic power = 16 W, and sonication duratio*n = *100 s). Fluorescence images (20 × magnification) from (6) different mice at the cortex level where plaques are stained green; Αβ (1–16) and the antibody red; Αβ (1–40). Co-localization of antibodies (white circles) in the cortex confirmed the successful entry and specific binding of the Aβ (1–40) antibody

Indicative results of immunohistochemistry analysis of brain tissue sections from 5XFAD mice are presented in Figs. 6 and 7. Co-localization of red (injected Aβ (1–40) antibody) and green (Αβ (1–16) antibody) fluorescence in multiple brain regions of the sonicated hemisphere confirmed successful BBBD, as well as entry and binding of the injected Αβ (1–40) antibody to Αβ plaques. Indicative fluorescence images of brain sections at the level of the cortex for the various mice groups are shown in Fig. 6. As expected, the 5XFAD mice that were injected with saline following FUS + MBs (saline group) did not have any signs of the Aβ (1–40) antibody in their brain. Similarly, the antibody was not present in any of the brain sections of the mice injected only with the Aβ (1–40) antibody (antibody group), confirming the inability of the specific therapeutic agent to normally pass through the BBB. On the contrary, immunohistochemistry analysis of brain sections from the FUS + MBs plus Ab group showed entry of the Aβ (1–40) antibody in the brain parenchyma. Note that the control mouse was not injected with the antibody neither received FUS + MBs; it was just stained with the Aβ (1–16) and Aβ (1–40) antibodies to confirm the presence of amyloid plaques (green & red) in the cortex. These findings qualify the selected treatment protocol (50 μL antibody & 1 MHz pulsed FUS with 10 ms burst length and 1% DF at 16 W for 100 s) as an efficient BBBD method for the delivery of the specific anti-Aβ antibody into the mouse brain. The repeatability of obtained results was investigated in ten mice, which all showed successful antibody entry and specific binding to plaques. Indicative brain sections from six mice are shown in Fig. 7 revealing co-localization of antibodies (white circles) in the cortex.

## Discussion

FUS in combination with MBs has been confirmed by numerous studies [10, 32, 33] as an effective method for overcoming the BBB to deliver exogenous therapeutic agents to the brain at present. AD is the most common cause of dementia [14] with Aβ immunotherapy belonging to the most promising therapeutics to alter its course [27]. Several antibodies such as Aducanumab and Lecanemab/BAN2401 were used for targeting Aβ at different epitopes (3–7 and 1–16, respectively) in order to promote amyloid plaque clearance [34]. However, drug efficacy is low as only 0.1% of antibodies can pass the BBB [35]. This study aimed to investigate whether FUS-mediated BBB opening with an optimized protocol can be used to safely and efficiently deliver a specific anti-Aβ antibody; Aβ (1–40) into the brain of 5XFAD AD mice using a custom-made FUS positioning device. The specific antibody is directed against the amyloid peptides Aβ (1–40) that represent the most abundant Aβ isoform in the AD brain [27], thus promoting plaque clearance. Unfortunately, its entrance in the brain is prohibited by the BBB due to its large molecular weight (150 KDa).

To our knowledge, this is the second study to report the use of the anti-Aβ antibody Aβ (1–40). In fact, there is a previous study that examined the feasibility of delivering this antibody into 3 mice by FUS-mediated BBBD. Authors report a three-fold increase in fluorescence intensity of the antibody staining in brain regions treated with MRgFUS (in comparison to non-sonicated regions), with the H&E staining revealing hemorrhage in the sonicated brain tissue [28]. We have verified these preliminary findings in a larger mice population (*n = *10) showing that FUS-mediated BBBD facilitates antibody penetration into the brain. In this study, non-sonicated mice showed zero fluorescence intensity indicating complete absence of the exogenous antibody in the examined brain tissue. It is worth mentioning that the absence of fluorescence intensity in the non-sonicated mice confirms that the anaesthetic (Avertin) used during the experimental procedure did not affect the BBB permeability, whereas other anaesthetics such as propofol affect BBB permeability [36]. Additionally, our current results go beyond previous findings further demonstrating that the use of an optimized protocol allows for efficient BBBD and delivery of the specific antibody without any tissue damage and probably the use of a smaller antibody dose (half compared to the dose used previously [28]). However, this requires further investigation.

The first experimental part was carried out in WT mice (*n = *32) and was devoted to selecting the acoustic power/pressure for optimized BBBD. Notably, at sufficiently high acoustic pressure, the administered MBs begin to oscillate stably causing transient increase of permeability in the targeted area while above a threshold of pressure inertial cavitation occurs where MBs collapse violently [37, 38]. In the former case, the endothelial ligaments recover completely within a few hours post-sonication [39]. Inertial cavitation is responsible for the majority of adverse effects observed with this strategy, such as micro-hemorrhages [38]. Therefore, the various acoustic pressure levels were tested both in terms of BBBD extent using the EB dye method and sonication-related tissue effects using H&E staining.

The EB dye technique allowed visual confirmation of BBBD with the naked eye directly after brain exposure and ease assessment of BBBD using a fluorescence microscope. A potential limitation of this methodology is that it does not provide any quantitative information on the magnitude of BBBD [40]. The FUS + MBs treated mice showed higher levels of EB dye in all examined brain areas, whereas for the control mouse (EB only) the dye remained in the extracellular matrix. The in situ peak pressure amplitude of 0.5 MPa (16 W acoustic power) applied at a frequency of 1 MHz in the presence of MBs (5 μL) was selected as offering safe and efficient BBBD and employed in follow-up studies involving the antibody. The results of H&E histology revealed no structural damage and no signs of hemorrhage in none of the sonicated hemispheres.

These results are consistent with what has been found in previous state-of-the-art studies. In fact, pressure levels of up to 0.5 MPa have been previously proposed by Hynynen et al. [39] as suitable for consistent and safe BBBD in rabbits, where the observed side effects were mostly limited to few tiny extravasations of red blood cells. Above that value and up to 1.4 MPa more severe effects such as hemorrhages and mild damage to the brain parenchyma were observed. Herein, none of the tested focal pressures ranging from 0.3 to 0.6 MPa (in situ*)* showed evidence of FUS-related effects on tissue integrity. The efficiency of the selected pressure level (0.5 MPa) to disrupt the BBB with negligible effects on brain tissue has been confirmed by other studies as well, with McDannold et al. [41, 42] reporting an estimated minimum threshold of 0.36 MPa for BBB opening. When comparing results, it must be pointed out that similar pulsed FUS parameters (10 ms burst length at 1 Hz repetition frequency) were employed in these studies, but a quite smaller frequency of 0.7 MHz.

Transgenic mouse models of AD constitute the main research tool in such studies since they are inexpensive, reproducible, and exhibit abundant plaque load. Herein, 5XFAD mice were bred for the antibody study (*n = *21). This is an excellent model since it recapitulates major features of the AD amyloid pathology at a very early age with a rapid amyloid beta plaque formation and severe gliosis [43, 44]. This is advantageous compared to other mouse models of AD that develop the pathology at a slower rate [45, 46]. It should be though noted that the absence of tau pathology that is a hallmark of AD can be a limitation of this model. Given that the interplay of Aβ and tau plays a major role in the development and acceleration of the disease, the disease mechanisms are not fully demonstrated [47].

The combined treatment involved retro-orbital injection of 50 μL of Aβ (1–40) antibody (2.85 mg), which is half the amount used in a previous study [28], and 5 μL of MBs (1 × 10^6^ MBs) followed by pulsed FUS (16 W) at 1 MHz. Retro-orbital injection was used as an alternative to tail vein intravenous administration. Based on the literature, there is no difference in the drug delivery, absorption or pharmacokinetic activity of therapeutic agents such as drugs or antibodies [48, 49]. Following FUS treatment, the mouse was left 4 h before it was sacrificed, which is considered the reliable post-treatment time window during which the BBB remains open [50, 51], to allow the maximum amount of antibody to enter and distributed in the brain. Immunohistochemistry analysis of brain tissue sections confirmed that the antibody cannot normally enter the brain parenchyma. Specifically, no fluorescent was observed in the microscope indicating absence of the antibody when administered alone owing to its prohibitively large molecular weight of 150 kDa. A single treatment with the selected sonication protocol (1 MHz pulsed FUS with 10 ms burst length, 1% DF, 16 W power, and 100 s duration) allowed the injected Aβ (1–40) antibody to enter the brain. In merged images, co-localization the Aβ (1–40) and Aβ (1–16) antibodies confirmed the presence of cortical plaques, successful trans-BBB entry of the injected anti-Aβ antibody in the sonicated brain, as well as the specificity of the Aβ (1–40) antibodies to bind to the amyloid plaques. The results showed excellent consistency and reproducibility of BBBD and FUS-mediated antibody delivery by single sonication in the treated hemispheres of all mice (*n = *10, FUS + MBs plus Ab group). It is expected that antibody binding to amyloid plaques will enhance their clearance from the brain by facilitating recognition and uptake by glial and peripheral immune cells, thus leading to reduction of amyloid (1–40) loading and subsequently inhibiting toxic oligomerization of Aβ [52].

This is a preliminary study that was focused on the feasibility of safely and efficiently delivering the Αβ (1–40) antibody into the mouse brain following BBB opening by FUS. Although the antibody was widely distributed through the sonicated brain and bound to Aβ plaques, it remains unclear to which degree the selected antibody amount promotes plaque clearance and positive cognitive effects nor whether the antibody amount can be further decreased. Accordingly, longer-term studies are required to assess the effects of the antibody and dose on suppressing AD pathology, which may require repeated treatments.

The positioning device employed in the study was proven an ergonomic tool for trans-cranial FUS applications in mice. The special design of the system allowed attaching the water-filled cone to the mouse head with visual confirmation of proper coupling following easy targeting with the assistance of the laser system. The suitability of the single element FUS transducer of 1 MHz for the particular application of transcranial BBBD in rodents was demonstrated, being in agreement with other field studies where 1 MHz burst FUS was predominantly selected for similar applications [9, 53–55]. Since this was a feasibility study, we did not attempt targeting a specific brain region. A global targeting approach was instead used where the beam was focused in the center of the left hemisphere. The blue dyed area in the perfused brain slice of Fig. 4 covers almost the entire targeted hemisphere. This is reasonable since the beam of the selected transducer is wide with focal point dimensions (≈ 2.5 mm lateral diameter) comparable with the hemisphere dimensions. Of course, the extend of BBBD and EB extravasation depend on multiple other factors, such as the applied pressure and burst duration [56], as well as the type and dose of MBs [57]. Notably, the connectivity of the brain and the changes in the local environment (e.g., blood flow) after the FUS treatment might also contribute to this phenomenon. Generally, as evidenced by the extend of EB dye extravasation, FUS of 1 MHz applied with the specific transducer and proposed sonication parameters affected almost the entire targeted hemisphere, also provided the small size of the mouse brain. Follow up studies may use ultrasonic sources with stronger focusing and account for such parameters to enable a more specific delivery of the antibody in brain regions of interest.

## Conclusions

Overall, the study findings demonstrated that the Αβ (1–40) antibody that is normally hampered by the BBB can efficiently and safely enter the brain parenchyma and bind to Aβ plaques of the 5XFAD mouse model of AD by FUS + MBs-mediated BBB opening with the proposed optimized protocol. Follow-up studies will examine the effects of this antibody on Aβ clearance and plaque load reduction, as well as whether repeated treatments can impart significant positive effects on cognition. These results hold promise for the development of disease modifying therapies for AD patients via the non-invasive anti-Aβ antibody delivery in future clinical applications.

## Data Availability

All data generated or analysed in the present study are available from the corresponding author on reasonable request.
